# Wildly Growing Asparagus (*Asparagus officinalis* L.) Hosts Pathogenic *Fusarium* Species and Accumulates Their Mycotoxins

**DOI:** 10.1007/s00248-015-0717-1

**Published:** 2015-12-21

**Authors:** Łukasz Stępień, Agnieszka Waśkiewicz, Monika Urbaniak

**Affiliations:** Department of Pathogen Genetics and Plant Resistance, Institute of Plant Genetics, Polish Academy of Sciences, Strzeszyńska 34, 60-479 Poznań, Poland; Department of Chemistry, Poznań University of Life Sciences, Wojska Polskiego 75, 60-625 Poznań, Poland

**Keywords:** Fumonisins, Fungal plant pathogens, Molecular identification, Natural *Fusarium* populations, Trichothecenes, Zearalenone

## Abstract

*Asparagus officinalis* L. is an important crop in many European countries, likely infected by a number of *Fusarium* species. Most of them produce mycotoxins in plant tissues, thus affecting the physiology of the host plant. However, there is lack of information on *Fusarium* communities in wild asparagus, where they would definitely have considerable environmental significance. Therefore, the main scientific aim of this study was to identify the *Fusarium* species and quantify their typical mycotoxins present in wild asparagus plants collected at four time points of the season. Forty-four *Fusarium* strains of eight species—*Fusarium acuminatum*, *Fusarium avenaceum*, *Fusarium culmorum*, *Fusarium equiseti*, *Fusarium oxysporum*, *Fusarium proliferatum*, *Fusarium sporotrichioides*, and *Fusarium tricinctum*—were isolated from nine wild asparagus plants in 2013 season. It is the first report of *F. sporotrichioides* isolated from this particular host. Fumonisin B_1_ was the most abundant mycotoxin, and the highest concentrations of fumonisins B_1_–B_3_ and beauvericin were found in the spears collected in May. Moniliformin and enniatins were quantified at lower concentrations. Mycotoxins synthesized by individual strains obtained from infected asparagus tissues were assessed using in vitro cultures on sterile rice grain. Most of the *F. sporotrichioides* strains synthesized HT-2 toxin and *F. equiseti* strains were found to be effective zearalenone producers.

## Introduction

*Asparagus officinalis* L. is a seasonal crop of high economic importance in the countries of Central and Western Europe [1, 2]. With perennial growth habit, asparagus plants are prone to be infected by fungi, acting rather like endophytes or opportunistic plant pathogens than biotrophic microorganisms. Asparagus has been regarded as one of the most versatile hosts for fungi, likely to be colonized by various *Fusarium* species [3]. *Fusarium oxysporum* and *Fusarium proliferatum* are usually the most commonly occurring species in cultivated plants, although *Fusarium avenaceum*, *Fusarium culmorum*, and *Fusarium solani* have also been identified in naturally infected asparagus spears [4–7]. Furthermore, despite symptomless type of growth often reported for many *Fusarium* species, many of them are able to produce mycotoxins in plant tissues [8], which influence physiology of the host plant [9]. It has been reported that trichothecenes, zearalenone, and fumonisins are among *Fusarium*-produced metabolites that are the most harmful when consumed with food and feed by animals and humans [10]. *F. proliferatum* is one of the most effective fumonisin B producers [11, 12] and *F. culmorum*, *Fusarium graminearum*, and *Fusarium cerealis* are able to produce large amounts of group B trichothecenes (either deoxynivalenol or nivalenol), and, along with *Fusarium equiseti*, of zearalenone [13–15]. On the other hand, *Fusarium sporotrichioides* and *Fusarium poae* synthesize a range of group A trichothecenes [16, 17]. Besides fumonisins and, possibly, trichothecenes, moniliformin and beauvericin appear as the most likely present metabolites in asparagus plants [18]. The fungal presence in the basal part of spears is usually more frequent than in the apical part, suggesting that the fungus can spread inside the plant colonizing particularly heavily the underground plant parts—crowns and roots [18]. Similarly to other perennial crop species colonized by *Fusarium* species [19–21], the mycotoxin-contaminated asparagus plants, when consumed, pose a serious risk of mycotoxin intake. Therefore, continuously updated knowledge of the physiology and mycotoxicity of fungal community present in perennial crops becomes essential to avoid the risk of the exposure to mycotoxin-contaminated foodstuff. Moreover, fungicide control of the crop plant’s infection is often difficult, as the endophyte-acting species cause no disease symptoms and host plant response to fungal infection can be significantly changed, as it was already shown for maize [22]. Although the issue has not yet been studied well for asparagus plants, an assumption has been made that some of the mechanisms can be similar in different host species. The role of the mycotoxins synthesized by the endophytes in host plant tissues appears to be essential and, yet, has not been proven. Consequently, it definitely would be interesting to reveal the fungal communities in wild growing plants compared to the typically present in cultivated asparagus plants [4–7]. To answer all those questions, the base of the present study was founded, in which several wildly growing asparagus plants were monitored for the presence of *Fusarium* species and accumulation of their respective mycotoxins. Thus, the main scientific aims of the research were (a) to identify the *Fusarium* species and their typical mycotoxins present in plants collected from a long-abandoned asparagus orchard, (b) to compare the fungal communities and mycotoxins quantified in plant tissues collected at four time points of the season (May–October 2013), and, finally, (c) to analyze the amounts of mycotoxins synthesized by individual strains obtained from infected asparagus tissues, using in vitro tests.

## Materials and Methods

### Wild Asparagus Spears Collection

**Fig. 1:**
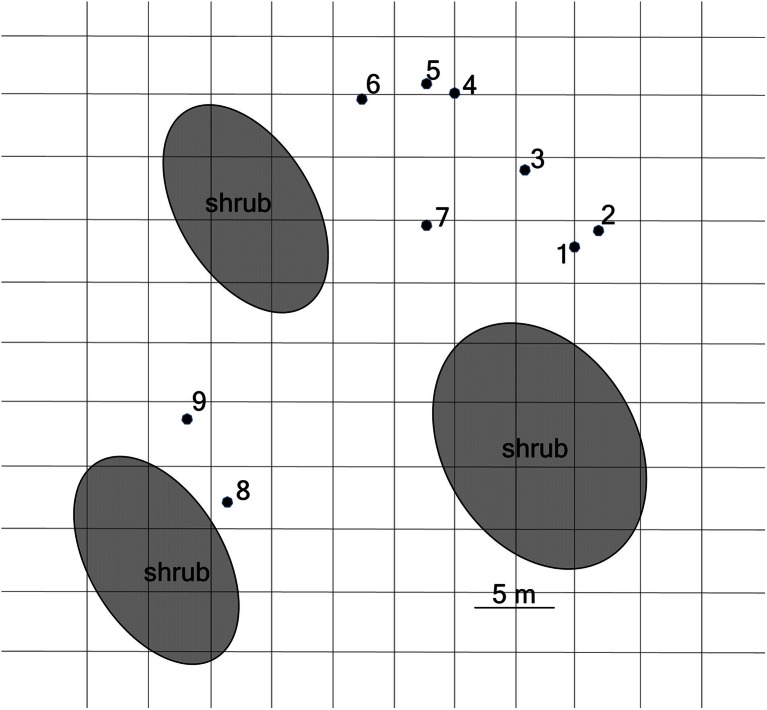
Schematic map of the area of the growth of nine asparagus plants used in the study

Wildly growing asparagus plants used in this study included nine individuals growing in an abandoned fruit orchard of about 500 m^2^ (Fig. 1) that has not been cropped for over 20 years and makes up for a large wasteland in Poznań, Central Poland. As the area is not used for any crop production, no agricultural influences are being acting nor crop protection techniques are being used. Thus, the plants can be considered as wild, avoiding any of the agricultural pressures. Spears (one piece per plant) have been collected continuously through 2013 season (May–October), i.e., from the start of the vegetation season until the beginning of winter senescence of the plants. Plants were sampled four times: in May, June, July, and October. All of the spears collected did not show visible symptoms of fungal infection, except for some snail damages and partial discoloration and lesions of those gathered in October, likely resulting from lignification process already started. All spears collected were subjected to the isolation of *Fusarium* fungi and quantification of fumonisins. Where the plant samples were large enough (5 g of fresh weight for each toxin analyzed), the identification and quantification of other mycotoxins have been performed: moniliformin, beauvericin and enniatins, zearalenone, and HT-2 toxin as a representative of the group A trichothecenes. Additionally, mycotoxin contents in the crowns samples of three selected plants were tested as well as in the fruit of only one plant (the remaining plants failed to form fruit in 2013 season).

### *Fusarium* Strains Isolation, Media, and Culture Conditions

Prior to fungal strain isolation, all plant samples were rinsed with sterile water to remove mineral remnants and surface-sterilized with 70 % ethanol to eliminate microbiological contaminants. Basal parts of the spears were cut into small pieces (three 5-mm slices of each spear) and plated aseptically on the potato dextrose agar (PDA, Oxoid, Basingstoke, UK) medium for 5–7 days at 20–25 °C and 12-h photoperiod. Then, growing mycelia of individual *Fusarium* strains were purified using Leslie and Summerell manual [23] and maintained in pure cultures for 7 days on PDA medium for genomic DNA extraction. All pure strains are maintained at the KF *Fusarium* Strains Collection at the Institute of Plant Genetics, Polish Academy of Sciences, Poznań, Poland. Number of strains isolated from individual wild asparagus plants has been summarized in Table 1.

**Table 1:** *Fusarium* strains isolated from nine asparagus plants used in the study; the samples were collected at four time points of 2013 season

Plant no.	Date of spears collection			
	May 10th	Jun 10th	Jul 15th	Oct 11th
#1	*F. proliferatum* (KF 3792) *F. sporotrichioides* (KF 3791) *F. tricinctum* (KF 3795)	*F. proliferatum* (KF 3797) *F. oxysporum* (KF 3830)	*F. proliferatum* (KF 3829)	none
#2	*F. culmorum* (KF 3798) *F. equiseti* (KF 3800) *F. proliferatum* (KF 3799)	*F. tricinctum* (KF 3789) *F. equiseti* (KF 3832)	*F. proliferatum* (KF 3801) *F. avenaceum* (KF 3803)	none
#3	*F. equiseti* (KF 3813) *F. sporotrichioides* (KF 3804)	*F. sporotrichioides* (KF 3813)	n/a	*F. sporotrichioides* (KF 3836)
#4	*F. oxysporum* (KF 3823) *F. proliferatum* (KF 3828) *F. sporotrichioides* (KF 3824)	*F. sporotrichioides* (KF 3825)	n/a	*F. sporotrichioides* (KF 3838)
#5	*F. acuminatum* (KF 3811) *F. proliferatum* (KF 3808) *F. sporotrichioides* (KF 3812)	*F. sporotrichioides* (KF 3835) *F. proliferatum* (KF 3810)	n/a	*F. equiseti* (KF 3839) *F. sporotrichioides* (KF 3840)
#6	*F. oxysporum* (KF 3790)	*F. oxysporum* (KF 3805)	*F. proliferatum* (KF 3806)	*F. oxysporum* (KF 3807) *F. proliferatum* (KF 4843) *F. proliferatum* (KF 3844)
#7	n/a	*F. equiseti* (KF 3816) *F. oxysporum* (KF 3817) *F. sporotrichioides* (KF 3818)	n/a	*F. proliferatum* (KF 3846) *F. sporotrichioides* (KF 3847)
#8	*F. proliferatum* (KF 3820)	n/a	n/a	*F. proliferatum* (KF 3848)
#9	n/a	*F. proliferatum* (KF 3822)	*F. proliferatum* (KF 3827)	none
Total	16	13	5	10
				44

*n/a* no new spears available

### DNA Extraction and Molecular Species Identification

Forty-four *Fusarium* strains isolated and purified from wild asparagus plants were identified to the species level using molecular techniques following the procedure validated earlier [24] and outlined below. Mycelia of individual strains were harvested from the PDA plates after 7 days of incubation. Genomic DNA was extracted using a hexadecyltrimethylammonium bromide (CTAB) method described previously [25], and the DNA extracts were stored at −20 °C. Species identification was done on the basis of the sequence analysis of a variable fragment of the translation elongation factor 1α (*tef-*1α). Fragments of all strains were PCR-amplified using the following primer pair: for Ef728M, 5′-CATCGAGAAGTTCGAGAAGG-3′, and for Tef1R, 5′-GCCATCCTTGGAGATACCAGC-3′. The primers were successfully validated on wide range of *Fusarium* species during previous studies [14, 26]. PCRs were done in 20-μL aliquots using C-1000 thermal cyclers (Bio-Rad, Hercules, CA, USA). Each reaction tube contained 0.4 μL of Phire II HotStart Taq DNA polymerase (Thermo Scientific, Espoo, Finland), 4 μL of 5× PCR buffer, 12.5 pmol of forward/reverse primers, 2.5 mM of each dNTP, and about 20 ng of fungal DNA. PCR conditions were as follows: 30 s at 98 °C; 35 cycles of 5 s at 98 °C, 5 s at 63 °C, and 15 s at 72 °C; and 1 min at 72 °C. Amplicons were electrophoresed in 1.5 % agarose gels (Invitrogen) with Midori Green dye (Nippon Genetics Europe Gmbh).

For sequence analysis, PCR-amplified DNA fragments were purified with exonuclease I (Thermo Scientific) and shrimp alkaline phosphatase (Thermo Scientific) using the following programme: 30 min at 37 °C and 15 min at 80 °C. Both strands were labeled using EF-728 M and Tef1R primers and the BigDyeTerminator 3.1 kit (Applied Biosystems, Foster City, CA, USA), according to Stępień et al. [14] and precipitated with 96 % ethanol. Sequence reading was performed using Applied Biosystems equipment. Sequences of PCR fragments were aligned using BLASTn algorithm to the sequences of reference strains belonging to individual *Fusarium* species, deposited in the GenBank Database. They were assigned to the reference species, of which both sequence coverages and nucleotide identities were matching the query with 99–100 %. All sequences obtained for 44 individual strains of eight *Fusarium* species isolated from wild asparagus plants have been deposited in the GenBank (NCBI) Database under Accession Numbers: KP729059-KP729102.

### Mycotoxins Analyzed in Asparagus Spears and Fungal Strain Cultures

Extraction, purification, and HPLC analysis for each mycotoxin in samples of wild asparagus plants and culture samples were done according to detailed procedures described earlier by Stępień and Waśkiewicz [15, 21, 26, 27]. For the quantification of mycotoxin synthesized, each strain was inoculated onto a flask containing 50 g of sterilized long-grain rice and 12.5 mL of water, following a procedure described by Stępień et al. [24].

The chromatographic system used to determinate mycotoxins level consisted of Waters 2695 high-performance liquid chromatography (HPLC) unit (Waters, Milford, USA) coupled with Waters 2996 Photodiode Array Detector and Waters 2475 Multi λ Fluorescence Detector. Empower™ 1 software was used for data processing (Waters, Milford, USA).

Standards of mycotoxins (fumonisins FB_1–3_, enniatins A, A_1_, B, B_1_, beauvericin, moniliformin, zearalenone, and HT-2 toxin) were purchased with a standard grade certificate from Sigma-Aldrich (Steinheim, Germany). The standard solutions of each mycotoxin (in ng/μL) were prepared in methanol. Organic solvents (HPLC grade) and all the other chemicals were also purchased from Sigma-Aldrich (Steinheim, Germany). Water for the HPLC mobile phase was purified using a Milli-Q system (Millipore, Bedford, MA, USA).

Detection limits for individual mycotoxins were as follows: 15 ng/g for beauvericin, 10 ng/g for enniatin A and A_1_, 8 ng/g for enniatin B and B_1_, 3 ng/g for zearalenone and HT-2 toxin, and 10 ng/g for moniliformin, and fumonisins FB_1_, FB_2_, and FB_3_ [15, 21, 27, 28]. Because the amounts of FBs in spear samples are usually too low to be detected, fumonisins were concentrated using vacuum evaporation of the extracts and re-dissolving them in methanol. Thus, the results presented in Table 3 may appear to be lower than the detection limit, because they were calculated for fresh weight amounts.

## Results

### *Fusarium* Species Isolated from Wild Asparagus Plants

*Fusarium* community found in nine wild asparagus plants during 2013 season was assessed. The number of *Fusarium* species in individual asparagus plants observed through four time points has been summarized in Table 1.

**Fig. 2:**
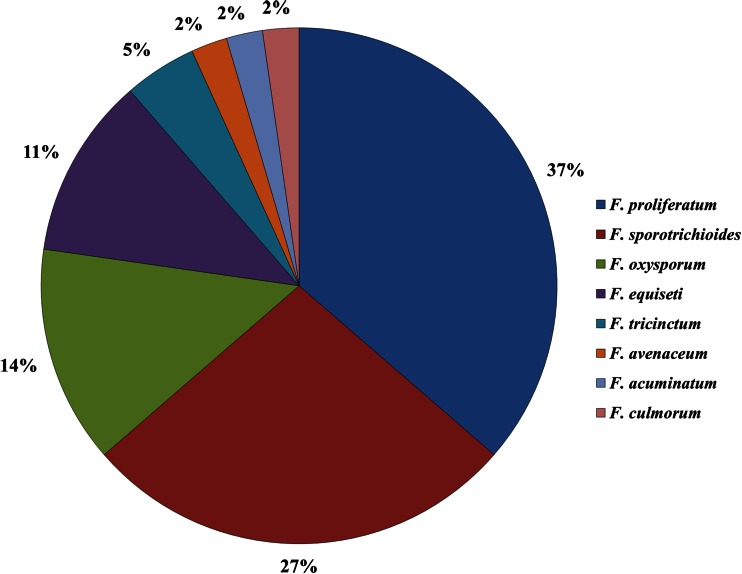
Overall frequencies of eight *Fusarium* species (represented by 44 isolates in total) identified in nine asparagus plants collected at four time points (see Table 1 for details)

Forty-four *Fusarium* strains were isolated, purified, and characterized, originating from nine asparagus plants from which spears were collected through one whole vegetation season 2013. Eight *Fusarium* species: *Fusarium acuminatum*, *F. avenaceum*, *F. culmorum*, *F. equiseti*, *F. oxysporum*, *F. proliferatum*, *F. sporotrichioides*, and *Fusarium tricinctum* were identified using molecular analyses based on sequence analysis of *tef-*1α gene. Overall frequencies of the species isolated from all plants from the orchard across the vegetation season are shown on Fig. 2. *F. proliferatum* and *F. sporotrichioides* were the prevailing species, and this is the first report of the latter species to be isolated from this host plant. Consistently, the same *Fusarium* species were frequently isolated from different spears of the same plants, however, it was also demonstrated that some species have been isolated only once during the season (Table 1). Strains of the same species isolated from a single plant but at different time points were regarded as independent strains, although possibly being identical. Apart from *Fusarium* species, *Alternaria*, *Epicoccum*, *Mucor*, and *Trichoderma* fungi were frequently occurring on asparagus plants tested (results not shown).

### Mycotoxins Analyzed in Asparagus Spears

Different groups of mycotoxins were found in plant samples after tissue portions have been used for the isolation of *Fusarium* strains: fumonisins, moniliformin, beauvericin, and enniatins A, A_1_, B, and B_1_ (Table 2). The highest concentrations of fumonisins were found in the spears collected in May, at the very beginning of the vegetation season. Later on, the amounts of mycotoxin accumulated dropped down substantially. Moreover, the differences were recorded in individual toxin concentrations for samples of the apical and basal stem parts. Interestingly, no uniform pattern could be observed, as some of the stems contained more mycotoxin in the apical part, while others in basal part of the stem (Table 2). The amounts of the HT-2 toxin measured for the spears with confirmed presence of *F. sporotrichioides* were below detection limits (data not shown).

**Table 2:** Mean values (±standard deviations) for mycotoxins in fresh asparagus plant tissues collected at four time points through May–October 2013 in Central Poland (in ng/g)

Plant no.	Plant part	Mycotoxins (ng/g)								
		FB_1_	FB_2_	FB_3_	Enn B	Enn B_1_	Enn A_1_	Enn A	MON	BEA
May										
1	Spear	3.27 ± 0.41	0.34 ± 0.04	0.21 ± 0.03	–	–	–	–	–	–
2	Top	9.33 ± 1.11	1.05 ± 0.13	nd	nd	nd	nd	nd	10.45 ± 1.27	103.64 ± 9.53
	Base	5.38 ± 0.47	nd	nd	20.16 ± 3.08	nd	nd	nd	nd	42.54 ± 3.74
	Spear	9.29 ± 0.85	1.08 ± 0.14	0.76 ± 0.08	–	–	–	–	–	–
3	Top	6.51 ± 0.72	0.38 ± 0.05	nd	nd	nd	43.49 ± 3.92	nd	nd	nd
	Base	5.21 ± 0.49	nd	nd	nd	nd	nd	nd	nd	47.57 ± 4.25
4	Spear	2.25 ± 0.18	0.14 ± 0.02	0.06 ± 0.01	–	–	–	–	–	–
5	Top	2.83 ± 0.33	nd	nd	nd	nd	nd	nd	15.96 ± 2.02	nd
	Base	4.02 ± 0.35	nd	nd	40.33 ± 5.12	nd	nd	nd	nd	nd
6	Spear	19.52 ± 2.02	0.98 ± 0.12	nd	–	–	–	–	–	–
8	Spear	2.87 ± 0.37	nd	nd	nd	nd	nd	nd	nd	24.77 ± 3.18
June										
1	Young spear	nd	nd	nd	–	–	–	–	–	–
	Crown	nd	nd	nd	–	–	–	–	–	–
	Green fruit	nd	nd	nd	–	–	–	–	–	–
	Spear	3.20 ± 0.28	2.07 ± 0.25	nd	nd	nd	23.35 ± 2.05	nd	nd	34.56 ± 4.21
2	Young spear	18.15 ± 2.01	0.17 ± 0.02	nd	–	–	–	–	–	–
	Crown	14.82 ± 1.55	1.37 ± 0.15	0.96 ± 0.11	–	–	–	–	–	–
	Spear	1.34 ± 0.14	nd	nd	–	–	–	–	–	–
3	Young spear	0.11 ± 0.02	nd	nd	–	–	–	–	–	–
	Crown	nd	nd	nd	–	–	–	–	–	–
	Spear	nd	nd	nd	–	–	–	–	–	–
4	Spear	6.51 ± 0.81	0.46 ± 0.06	nd	nd	nd	nd	nd	nd	nd
5	Spear	2.62 ± 0.36	nd	nd	54.73 ± 4.87	nd	nd	nd	nd	21.31 ± 2.23
6	Spear	nd	nd	nd	nd	nd	nd	nd	nd	nd
7	Spear	nd	nd	nd	nd	nd	23.22 ± 3.16	nd	nd	20.39 ± 1.84
9	Spear	4.14 ± 0.34	0.36 ± 0.04	nd	nd	nd	15.02 ± 2.05	nd	nd	23.64 ± 2.25
	Top	7.23 ± 0.86	nd	nd	–	–	–	–	–	–
	Base	14.21 ± 1.77	0.84 ± 0.07	nd	–	–	–	–	–	–
	Top	9.36 ± 1.15	0.71 ± 0.08	nd	–	–	–	–	–	–
	Base	13.66 ± 1.40	1.02 ± 0.21	nd	–	–	–	–	–	–
July										
1	Top	0.79 ± 0.11	nd	nd	–	–	–	–	–	–
	Base	nd	nd	nd	–	–	–	–	–	–
4	Red fruit	27.37 ± 3.02	0.98 ± 0.10	nd	–	–	–	–	–	–
6	Spear	5.00 ± 0.54	nd	nd	–	–	–	–	–	–
7	Top	132.99 ± 15.83	6.74 ± 0.72	5.13 ± 0.55	–	–	–	–	–	–
	Base	26.09 ± 2.92	2.11 ± 0.30	nd	–	–	–	–	–	–
9	Top	nd	nd	nd	–	–	–	–	–	–
	Base	0.21 ± 0.01	nd	nd	–	–	–	–	–	–
October										
1	Spear	31.82 ± 3.66	2.15 ± 0.31	nd	nd	nd	47.29 ± 5.11	nd	nd	10.74 ± 0.98
2	Spear	5.55 ± 0.67	nd	nd	nd	nd	16.00 ± 2.07	nd	nd	nd
3	Spear	6.86 ± 0.75	nd	nd	–	–	–	–	–	–
4	Spear	1.07 ± 0.25	nd	nd	nd	nd	nd	nd	27.13 ± 3.14	27.10 ± 3.15
5	Spear	1.11 ± 0.17	nd	nd	–	–	–	–	–	–
6	Spear	13.50 ± 1.48	2.45 ± 0.19	nd	–	–	–	–	–	–
7	Spear	nd	nd	nd	–	–	–	–	–	–
8	Spear	80.23 ± 8.11	8.14 ± 0.94	1.16 ± 0.13	–	–	–	–	–	–
9	Spear	12.79 ± 1.45	2.50 ± 0.41	nd	nd	nd	nd	nd	44.96 ± 4.32	nd

FB amounts have been re-calculated for the fresh weight amounts. Please refer to Table 2 for spear availability

*nd* below detection limit

### Mycotoxins Analyzed in Fungal Strain Cultures

Furthermore, the amounts of mycotoxins synthesized by individual *Fusarium* strains were analyzed using sterile rice grain cultures. Moderate levels of toxins have been found for most of the *F. proliferatum*, *F. equiseti*, and *F. sporotrichioides* strains (Table 3). Four out of 12 *F. sporotrichioides* strains (KF 3791, KF 3818, KF 3835, and KF 3840) did not synthesize the HT-2 toxin (group A trichothecene), and the ability to synthesize zearalenone was confirmed for all strains of *F. equiseti* and *F. culmorum*, the species known as casual producers of the respective mycotoxins (Table 3).

**Table 3:** Mycotoxin biosynthesis (mean values ± standard deviations) by 39 *Fusarium* isolates obtained from nine asparagus plants during the study (FBs given in μg/g, the remaining mycotoxins in ng/g)

Isolate	Species	FB_1_	FB_2_	FB_3_	Enns	MON	BEA	ZON	HT2
KF 3803	*F. avenaceum*	nd	nd	nd	27.11 ± 2.34 (Enn B) 15.44 ± 1.63 (Enn A_1_)	34.98 ± 4.11	nd	nd	nd
KF 3798	*F. culmorum*	nd	nd	nd	55.38 ± 4.95 (Enn B)	nd	nd	6831.32 ± 554.69	nd
KF 3800	*F. equiseti*	nd	nd	nd	nd	nd	nd	8050.45 ± 622.31	nd
KF 3816	*F. equiseti*	nd	nd	nd	nd	nd	nd	10054.83 ± 852.73	nd
KF 3832	*F. equiseti*	nd	nd	nd	nd	nd	nd	266.80 ± 30.18	nd
KF 3839	*F. equiseti*	nd	nd	nd	nd	nd	nd	5980.41 ± 416.36	nd
KF 3813	*F. equiseti*	nd	nd	nd	68.79 ± 7.17 (Enn B) 64.05 ± 5.37 (Enn B_1_) 131.19 ± 10.51 (Enn A_1_)	3351.38 ± 205.44	nd	13079.92 ± 1014.22	nd
KF 3790	*F. oxysporum*	nd	nd	nd	nd	45.21 ± 4.63	nd	nd	nd
KF 3805	*F. oxysporum*	nd	nd	nd	nd	532.84 ± 48.77	903.38 ± 81.47	nd	nd
KF 3817	*F. oxysporum*	nd	nd	nd	nd	1081.13 ± 95.23	nd	nd	nd
KF 3823	*F. oxysporum*	nd	nd	nd	nd	110.36 ± 9.88	nd	nd	nd
KF 3799	*F. proliferatum*	nd	nd	nd	nd	254.11 ± 21.13	63.85 ± 7.11	nd	nd
KF 3801	*F. proliferatum*	282.40 ± 25.16	81.37 ± 8.08	9.97 ± 1.13	nd	174.03 ± 18.45	47.66 ± 5.25	nd	nd
KF 3806	*F. proliferatum*	249.95 ± 22.83	59.57 ± 6.62	4.90 ± 0.62	nd	nd	nd	nd	nd
KF 3807	*F. proliferatum*	nd	nd	nd	nd	290.41 ± 30.55	nd	nd	nd
KF 3808	*F. proliferatum*	200.10 ± 19.84	53.35 ± 5.09	19.16 ± 2.23	nd	nd	nd	nd	nd
KF 3810	*F. proliferatum*	0.24 ± 0.02	0.11 ± 0.01	0.05 ± 0.01	nd	89.34 ± 10.07	63.57 ± 4.99	nd	nd
KF 3820	*F. proliferatum*	183.52 ± 20.15	39.45 ± 4.22	25.94 ± 3.00	nd	8151.55 ± 541.28	782.00 ± 60.52	nd	nd
KF 3822	*F. proliferatum*	332.48 ± 31.08	61.67 ± 5.93	17.90 ± 1.28	nd	284.11 ± 30.21	74.64 ± 5.88	nd	nd
KF 3827	*F. proliferatum*	325.38 ± 29.77	48.09 ± 4.27	8.91 ± 1.03	nd	nd	94.22 ± 10.08	nd	nd
KF 3828	*F. proliferatum*	383.87 ± 40.12	124.04 ± 11.67	12.97 ± 1.44	nd	1102.65 ± 81.52	nd	nd	nd
KF 3829	*F. proliferatum*	53.21 ± 5.76	4.54 ± 0.52	4.00 ± 0.45	nd	978.54 ± 100.08	231.05 ± 19.63	nd	nd
KF 3843	*F. proliferatum*	720.91 ± 69.93	211.85 ± 18.80	28.73 ± 3.42	nd	524.88 ± 42.16	nd	nd	nd
KF 3844	*F. proliferatum*	307.05 ± 29.55	79.66 ± 9.16	28.73 ± 2.26	nd	225.34 ± 15.96	147.68 ± 20.31	nd	nd
KF 3846	*F. proliferatum*	98.85 ± 10.18	22.98 ± 1.94	1.13 ± 0.17	nd	1025.88 ± 114.58	312.59 ± 25.64	nd	nd
KF 3848	*F. proliferatum*	337.49 ± 30.67	114.76 ± 15.68	2.54 ± 0.19	nd	496.37 ± 50.23	107.58 ± 8.77	nd	nd
KF 3792	*F. proliferatum*	363.20 ± 40.12	73.57 ± 7.11	7.86 ± 0.81	131.34 ± 10.89 (Enn B) 385.39 ± 35.82 (Enn A_1_)	2307.99 ± 185.12	404.66 ± 32.58	nd	nd
KF 3797	*F. proliferatum*	137.69 ± 15.08	71.81 ± 6.94	12.62 ± 1.15	58.65 ± 6.18 (Enn B_1_)	nd	nd	nd	nd
KF 3804	*F. sporotrichioides*	nd	nd	nd	nd	nd	nd	nd	62.17 ± 7.42
KF 3812	*F. sporotrichioides*	nd	nd	nd	nd	nd	nd	nd	51.02 ± 3.95
KF 3824	*F. sporotrichioides*	nd	nd	nd	nd	nd	61.27 ± 7.12	nd	67.24 ± 5.87
KF 3825	*F. sporotrichioides*	nd	nd	nd	nd	142.06 ± 12.17	nd	nd	90.11 ± 10.45
KF 3836	*F. sporotrichioides*	nd	nd	nd	nd	nd	nd	nd	79.54 ± 6.13
KF 3838	*F. sporotrichioides*	nd	nd	nd	nd	nd	nd	nd	101.88 ± 9.09
KF 3847	*F. sporotrichioides*	nd	nd	nd	nd	nd	nd	nd	47.59 ± 3.58
KF 3815	*F. sporotrichioides*	nd	nd	nd	90.02 ± 10.25 (Enn A_1_)	nd	213.53 ± 19.82	nd	33.12 ± 4.11
KF 3830	*F. tricinctum*	nd	nd	nd	nd	25.17 ± 2.44	nd	nd	nd
KF 3789	*F. tricinctum*	nd	nd	nd	89.02 ± 7.45 (Enn A_1_)	91.25 ± 10.11	nd	nd	nd
KF 3795	*F. tricinctum*	nd	nd	nd	283.52 ± 30.11 (Enn B) 378.41 ± 40.53 (Enn B_1_) 171.42 ± 20.00 (Enn A_1_) 101.4 ± 9.27 (Enn A)	7393.57 ± 254.33	547.79 ± 41.69	nd	nd

Five strains did not synthesize any of the metabolites studied—KF 3811 (*F. acuminatum*), KF 3791, KF 3818, KF 3835, and KF 3840 (*F. sporotrichioides*)—and were excluded from the Table

*nd* below detection limit

## Discussion

The knowledge about the occurrence of *Fusarium* pathogens on various perennial plants in temperate climate (including cultivated and non-cultivated species) has been rather scarce. Some of the reports published were focused on tropical fruit crops, including banana, date palm, mango, and pineapple [8, 19, 20, 28, 29] and ornamental plants [30]. *A. officinalis* L. is another perennial crop that can serve as a host to a range of fungal species, and the incidence and frequencies of individual *Fusarium* species on cultivated asparagus plants has been studied for many years [6, 7, 18, 31, 32]. *F. oxysporum* appeared as the most frequent pathogenic species causing crown rot and other *Fusarium-*related diseases in cultivated asparagus (Authors’ studies, unpublished), along with *F. proliferatum* [5]. Consequently, a lot of researchers’ attention was devoted to harmful mycotoxins synthesized by the fungi colonizing almost every plant tissue [1, 33]. However, to control the fungal contaminations of a perennial species is often a difficult task, since the infection process runs symptomless in many cases, although the plant tissues are by then heavily overgrown with fungal hyphae and contaminated with mycotoxins [4, 7]. Moreover, other specific factors (harvest time and frequency) affect the symptom occurrence on asparagus plants [34]. The worldwide distribution of crops colonized with fungi can have significant impact on ambient pathogen populations and introduction of new species into the local environment [8]. On the other hand, not much data is available on the fungal community present in wildly growing crop plants, i.e., those that are not protected, fertilized, and regularly harvested. Since the environment of the plant is different comparing to the cultivated plants (the proximity of rural plant and animal communities instead of a monoculture), it is also likely that mycobiota associated with these asparagus plants change, becoming endophytes rather than pathogens to endure inside the plant for a longer period of time. Similar relationships have already been noticed for asparagus plantations [34]. Therefore, the basic scientific aim of the present study was to evaluate the diversity of *Fusarium* spp. colonizing wildly growing asparagus plants. Research done during recent years (2009–2012) has proven the *F. oxysporum* to be consistently the main pathogen isolated from cultivated asparagus spears, followed by *F. proliferatum* (unpublished data) and just a few additional species (*F. equiseti* and *F. avenaceum*). The fact that the *Fusarium* community in wild plants is different from that of cultivated plants, may be as an outcome of the variation in environmental factors. Consequently, the secondary metabolites synthesized by the mycobiota have to be adjusted to such a specific lifestyle, or, alternatively, it can play different roles than just influencing the infection of the plant.

Nine plants were monitored for the presence of young spears in 1-month intervals. Additionally, green and ripe (red) fruit, as well as three crown samples, were screened for mycotoxin contamination and presence of *Fusarium* fungi. The species diversity found in non-cultivated asparagus plants was found to be higher than that reported for the marketable (i.e., cultivated) asparagus spears [4, 34]. This finding might suggest that asparagus plants can likely be infected with numerous *Fusarium* species and agricultural techniques can possibly limit their number to just two or three observed with the highest frequencies: *F. oxysporum* and *F. proliferatum* [5, 18]. Actually, the latter species was also present among the most frequently isolated fungi in the present study (Fig. 2 and Table 1). As a consequence, the contamination of plant tissues with fumonisins appears as very likely, since *F. proliferatum* often synthesizes FBs in infected plant tissues [1, 8, 35, 36]. Among 44 *Fusarium* isolates purified during the present study, while *F. proliferatum* and *F. sporotrichioides* were the prevailing species, only six *F. oxysporum* isolates were found (Table 1 and Fig. 2). Noteworthy, *F. sporotrichioides* was identified in asparagus plants with high frequency. To the best of our knowledge, it has not been reported as the pathogen of asparagus until now; therefore, more attention should be focused on cultivated plants to confirm its pathogenicity toward this crop. Thus, the identification of this species in wild asparagus appears as one of the most important findings of the present study, since the species is a well-known producer of the most toxic *Fusarium* metabolites, namely, the T-2 and HT-2 toxins [37] and the possible risk of contamination of asparagus spears with those compounds would pose a serious risk to the consumers. It is possible that weather conditions were favorable for *F. sporotrichioides* in this particular season, as it has also been frequently identified in tissues of other crop species, like *Triticum durum* (authors’ data, unpublished). Nevertheless, the species was identified in 2014 season in wild asparagus plants collected from other localities of Poland (data not shown); therefore, it should be considered as at least associated with this crop.

The diversity of *Fusarium* species in field-grown asparagus plants was usually limited to few species [1, 5–7, 31, 32]. Concerning individual plants, the incidence of *Fusarium* species varied significantly during the season, while from the plants #8 and #9, only *F. proliferatum* was isolated, and from plants #2 and #5, five and four different species were derived in total, respectively (Table 1). It seems that the incidence and prevalence of individual *Fusarium* species in the overall population are not constant and may change rapidly and continuously. On the other hand, fungi living as endophytes can be found in the same plants throughout the season. All species but one were isolated at the beginning of the season (May), while at the end (October), only few were isolated (Table 1). Definitely, additional research using dedicated approaches is needed to reveal the factors influencing those changes.

Fumonisins, associated with *F. proliferatum* as the main producer, have been the first mycotoxins of concern for many years, when asparagus plants were analyzed. Also in the present study, fumonisins B_1_–B_3_ were the most abundant mycotoxins found in virtually all plant samples. Moniliformin and cyclic peptide toxins (beauvericin and enniatins) were quantified at lower concentrations and frequencies (Table 2). Although mycotoxins were present in asparagus plants throughout the whole season, the highest levels of toxins were measured at the beginning of vegetation season (samples collected in May), particularly for the plants #1 and #2. Contradictory, the crowns of plants #1 and #3 contained no detectable FBs, despite the mycotoxins were present in the spears of the respective plants (Table 2). The presence of the fungus does not necessary mean the mycotoxin contamination. Moreover, in some plants sampled in October, there were fumonisins present, although no fungal strain has been isolated at that time (Tables 1 and 2). The absence of fungal strains late in the season is not surprising as the metabolic activity of most organisms is much lower than in the Spring (May). Thus, the latest spears may be sometimes not colonized by fungus, although the toxin may be spread along the vascular system.

Noteworthy, more toxins were usually present in the apical parts of the spear, than in the basal parts (Table 2), which could be likely explained by more intensive growth of the fungus in rapidly proliferating tissues. To confirm this hypothesis, however, additional experiments should be performed, measuring quantitatively fungal biomass in plant tissues (e.g., by ergosterol contents analysis or qPCR analyses). The level of mycotoxin synthesized by the pathogen highly depends on metabolic activity of the fungus, which in turn is related to a number of environmental factors (like photoperiod and temperature). These factors may be responsible for high FB amounts at the beginning of growing season (May). Subsequent decrease in the samples of respective plants is probably the result of lower activity of the pathogen than in the Spring. Few measured concentrations of FBs found in plant samples (Table 2) were higher than these reported for cultivated plants [4, 18]. Analyses of crown mycotoxin contamination late in the season could give more details about seasonal fluctuations in mycotoxin contents in asparagus plants.

Lower amounts of mycotoxins synthesized by *F. proliferatum* strains obtained from wild asparagus plants were observed (Table 3), particularly when compared to those measured for collection strains, originating from different host plant species [21]. Even *F. proliferatum* strains derived from cultivated asparagus plants produced more fumonisins than these of wild plants [38]. This finding might actually suggest possible existence of a distinct population of *F. proliferatum* colonizing wild asparagus plants that could be distinguishable from the genotypes present inside the cultivated asparagus plants. Based on preliminary molecular analyses, some intraspecific variance has been recorded inside the coding sequences of *tef-*1α gene and, particularly, in the *FUM1* gene (authors’ studies, unpublished), which usefulness for phylogenetic and polymorphism studies has already been proven on the intraspecific level [16, 38].

Another interesting finding was the incidence of only one of the four enniatins analyzed in the plant samples tested. The presence of a mixture of enniatin analogues in the naturally contaminated samples has been reported as frequent [13, 24, 39], and this rule was also true for rice cultures of various *Fusarium* species [26]. This coincidence of various enniatins was also found in cultures of *Fusarium* strains isolated from asparagus plants during the present study. The abilities to synthesize high amounts of moniliformin were confirmed for some of the isolates, and enniatins and beauvericin were generally less abundant. Similarly, the ability of *F. sporotrichioides* strains to synthesize HT-2 toxin was rather limited (Table 3). On the other hand, *F. equiseti* strains produced large amounts of zearalenone (Table 3), though not as high as some of the previously tested strains [40].

As a conclusion, it can be stated that populations of *Fusarium* species colonizing wildly growing asparagus plants appear as more diverse and with different species seem to prevail there (*F. proliferatum* and *F. sporotrichioides* dominate instead of *F. oxysporum* prevailing in plant material from commercial plantations). Consequently, the profiles of mycotoxins contaminating plant tissues vary as well, although fungal strains isolated from infected wild plants during the present study synthesized less mycotoxins than those originating from cultivated asparagus plants, as reported earlier [1, 21, 26]. Nevertheless, the environmental role of toxic fungal metabolites (e.g., their possible action as pesticides and/or insecticides or as the effector compounds of microorganisms competing for nutrients) remains to be revealed. The latter, however, would likely to be true, as usually more than one *Fusarium* species were found in the same plant throughout the vegetation season. Finally, the results obtained during the study implicate the possible important role of perennial plant species as the pathogen vectors, which definitely should be taken into account in future strategies aimed at controlling the economically and ecologically important plant diseases.
